# Health of children in Australian immigration detention centres: An analysis of the quarterly health reports from 2014 to 2017

**DOI:** 10.1111/jpc.15880

**Published:** 2022-01-18

**Authors:** Ryan Essex, Erika Kalocsányiová, James G Scott, Rosana Pacella

**Affiliations:** ^1^ Institute for Lifecourse Development The University of Greenwich London United Kingdom; ^2^ Mental Health Programme QIMRB Medical Research Institute Brisbane Queensland Australia; ^3^ Metro North Mental Health Service Brisbane Queensland Australia

**Keywords:** asylum seeker, health, human rights, immigration detention, refugee

## Abstract

**Aim:**

This study examines 3 years of child and adolescent health data from Australian onshore and offshore immigration detention centres from 2014 to 2017, quantifying the health presentation data of children and adolescents in Australian immigration detention and comparing rates between onshore and offshore detention.

**Methods:**

This study utilised the Quarterly Immigration Detention Health Reports over a period of 3 years. To compare onshore and offshore datasets, we calculated the rate of health events per quarter against the estimated quarterly onshore and offshore detention population of children. We ran a series of two‐proportion *z*‐tests for each matched quarter to calculate median *z* and *P* values for all quarters. These were used as an indicator as to whether the observed differences between onshore and offshore events were statistically significant.

**Results:**

The estimated number of children detained per quarter onshore ranged from 700 in 2014 (quarter 3) to 13 in 2016 (quarters 3 and 4); the estimated quarterly population of children in offshore detention ranged from 186 in 2014 (quarter 3) to 42 in 2017 (quarter 2). Children offshore had significantly higher rates of consultations with a mental health nurse (*z* = −1.96; *P* = 0.002), psychologist (*z* = −2.32; *P* = 0.01) and counsellor (*z* = −3.41; *P* < 0.001). As for reasons for presentation to general practitioners and psychiatrists, complaints related to skin (*z* = −1.97; *P* = 0.05), respiratory issues (*z* = −1.96; *P* = 0.05) and urological issues (*z* = −2.21; *P* = 0.03) were significantly higher amongst children detained offshore.

**Conclusions:**

Compared to children in the Australian community, children detained both onshore and offshore had greater health needs. Children offshore also presented more frequently with a range of complaints and accessed health services at higher rates than children detained onshore; this adds to growing evidence about the harms of offshore detention and detention more generally.

## What is already known on this topic

1. Australian immigration detention has a devastating impact on the health of children who are detained.
2. Growing evidence suggests that offshore detention has a far greater impact on health and wellbeing than onshore detention.

## What this paper adds

1. This article compares Australian government health data for children detained in onshore and offshore immigration detention, offering a direct comparison.
2. Children in onshore and offshore detention had substantial health needs, greater than that found in the general population.
3. Children detained offshore appeared to have greater health needs than those detained onshore.

Australia has maintained a policy of mandatory immigration detention for almost 3 decades. The detention of children, along with the impact of detention on health and wellbeing of individuals, has drawn persistent domestic and international criticism. While Australia has maintained centres on the mainland for over 3 decades, over the last 8 years, concerns about the detention of children have become increasingly pressing as Australia reopened offshore centres on Manus Island (Papua New Guinea) and Nauru. (For the remainder of the paper, we will refer to centres on the Australian mainland and Christmas Island as ‘onshore’ centres and Manus Island and Nauru as ‘offshore’ centres. From 2014 to 2017, Australia maintained several centres on the mainland, for more information and a map, see Essex.^1^) Since their opening, multiple riots, violence, sexual and physical abuse, self‐harm and suicides have occurred in these centres. These traumatic events have raised concerns about the welfare of detained children and their families.^1^ Since 2019, no children have been detained offshore and while the number of children detained onshore has decreased since 2015, children have been intermittently detained to this day. Additionally, all asylum seeker boat arrivals remain subject to offshore detention.

The Australian government has long withheld data that would give greater insight into these issues.^2^ Despite this, evidence has emerged suggesting that detention has a devastating impact on health, with high rates of mental health concerns amongst children and families. In a study of families detained in a remote onshore immigration detention centre, Steel *et al*.^3^ conducted telephone interviews without the knowledge of the immigration department. Results suggest that all adults and children met diagnostic criteria for at least one current psychiatric disorder. Retrospective comparisons indicated that adults displayed a 3‐fold and children a 10‐fold increase in psychiatric diagnoses subsequent to detention. Similar results were found by Mares and Jureidini^4^ who examined 16 adults and 20 children who were held in detention and referred to a child and adolescent mental health service. Of the 10 children aged 6–17 years that were examined all fulfilled criteria for both post‐traumatic stress disorder and major depression with suicidal ideation. Eight of the 10 children, including 3 pre‐adolescents, had made significant attempts at self‐harm. Seven had severe anxiety symptoms and half reported persistent severe somatic symptoms. The majority (80%) of pre‐school‐age children were identified with developmental delay or emotional disturbance. The authors concluded that the experiences in detention contributed to their mental health problems.

More recent evidence comes from the Australian Human Rights Commission who visited a number of onshore centres and Nauru while preparing the Forgotten Children Report.^5^ This report details the stories of a number of refugee and asylum seeker children and families held in detention centres and concluded that ‘[p]rolonged detention is having profoundly negative impacts on the mental and emotional health and development of children’. A number of authors further analysed the data gathered during this investigation. Young and Gordon^6^ re‐examined the data collected by the Australian Human Rights Commission for 25 onshore detention centres. One‐third of the children and adolescents had scores on the clinician‐rated Health of the Nation Child and Adolescent Outcome (HoNOSCA) scale that were equivalent to an Australian clinical population. (The HoNOSCA is a 15‐item questionnaire, developed for use in child and adolescent mental health services focusing on general health and social functioning.^7^) Mares^8^ also utilised these data to examine the mental health of children and their parents. Kessler Psychological Distress Scale (K10) scores indicated that 83% of adults and 85.7% of teenagers were likely to have a severe anxiety or depressive disorder. In children, Strengths and Difficulties Questionnaire (SDQ) scores suggest that 75.7% had a high probability of psychiatric disorder. Unsurprisingly, when compared with a matched sample in the Australian community, children in detention had worse mental health. Also utilising the SDQ, Zwi *et al*. found that children in detention presented with significantly more social, emotional and behavioural difficulties than those in the Australian community with scores of detained children equivalent to those in a clinical cohort.

While each of the above studies has made an important contribution to understanding the impact of immigration detention on children and their families, few have data where the mental health of those in onshore and offshore immigration detention can be compared. Since offshore detention was re‐introduced in 2013, there has been substantial anecdotal evidence to suggest it has a greater negative impact on the health and wellbeing of those detained.^10^

This study seeks to examine 3 years of child and adolescent health data from Australian onshore and offshore immigration detention centres from 2014 to 2017. This study has two overarching aims: first, to quantify the health and health‐care needs of children and adolescents in Australian immigration detention, outlining rates of access to health care, prescriptions and other key health indicators; second, to compare these rates between onshore and offshore detention with those in the Australian community. (While we refer to detention as ‘offshore’, children were only detained offshore on Nauru. A detailed discussion about the condition on Nauru is contained in the AHCR Forgotten Children Report).^5^

## Methods

### Data sources

In this study, we utilised the Australian government's Quarterly Immigration Detention Health Reports over a period of 3 years (from quarter 3, 2014, to quarter 2, 2017) for onshore and offshore detention (these reports are not available any earlier than these dates and the Australian government has not yet released reports beyond Q4 2017 offshore and Q4 2018 onshore). These reports contain data about the health and wellbeing of children, including complaints/presenting symptoms and number of appointments and hospitalisations, amongst other variables. These reports were either already publicly available (https://www.homeaffairs.gov.au/access-and-accountability/freedom-of-information/disclosure-logs) or obtained through Freedom of Information Requests sent to the Australian Department of Home Affairs. These reports only contain information about detention centres, not community detention. (The Australian government has detained children and families in a range of centres onshore; from the data discussed below, we cannot distinguish between centres. For more information on the types of detention in which children have been held, see Essex.^1^)

### Data, the detention population and variables

Data were entered manually by two authors (RE and EK), screened and cleaned. To compare onshore and offshore datasets, the data were transformed. To do this, we first had to estimate the quarterly detention population of children as available detention population statistics are cross‐sectional and calculated monthly (https://www.homeaffairs.gov.au/research‐and‐statistics/statistics/visa‐statistics/live/immigration‐detention). This was calculated by using the data contained within the quarterly health reports and is detailed in our Supporting Information.

After calculating the detention population for both onshore and offshore data, we estimated the rate per 100 children per quarter for key health indicators against the quarterly onshore or offshore detention population. We have relied on data reporting ‘unique individuals’ as opposed to ‘unique appointments’ per quarter. That is, rates reported below reflect the number of children per quarter who (on average) accessed services or were prescribed medication for example. Raw data from which these rates were calculated are detailed in our Supporting Information.

Given the detention population, and because quarter by quarter many of the same children were detained, data violated assumptions for independence of observations, limiting the significance tests that could be carried out. To overcome this, we opted to run a series of two‐proportion *z*‐tests for each matched quarter. After calculating *z* and *P* values for each quarter, we calculated median *z* and *P* values for all quarters and utilised this as an indicator as to whether the observed differences between onshore and offshore events were statistically significant.

This study included four variables: (i) the reasons why children presented to general practitioners (GPs) and psychiatrists; that is, the types of presentations seen that quarter by GPs and/or psychiatrists as a combined category; (ii) the number of consultations by health profession; that is, how many children attended appointments with various health professionals each quarter; (iii) number of children who were prescribed medication, and other health events where a comparison could be made including; and (iv) specialist referrals, chronic disease and disability rates and torture and trauma disclosures. Our raw data, along with a more detailed account of how the data were transformed and the variables included in this article, are detailed in the Supporting Information.

### Ethics approval

Ethics approval for this study was granted by the University of Greenwich, Human Research Ethics Committee (UREC/20.1.5.6).

## Results

### The detention population

Over the period of 2014 (quarter 3) to 2017 (quarter 2), there were 2450 observations made in onshore detention and 952 offshore. No data exist that indicate how many individual children this includes; however, given the often protracted nature of detention, it should be assumed, particularly for the offshore population that detained children presented across multiple quarters. The estimated total populations of children in onshore and offshore detention centres per quarter are reported in Table 1. Offshore, children were held on Nauru while onshore detention was distributed across a number of centres predominantly on mainland Australia. Children's demographic characteristics (gender and countries of origin) could not be extracted from the health reports.

**Table 1:** Estimated number of children detained per quarter in immigration detention centres onshore and offshore

	Onshore	Offshore
2014 Q3	700	186
2014 Q4	622	135
2015 Q1	455	103
2015 Q2	173	88
2015 Q3	153	92
2015 Q4	132	68
2016 Q1	110	54
2016 Q2	32	49
2016 Q3	13	45
2016 Q4	13	45
2017 Q1	33	45
2017 Q2	18	42
Total	2454	952

### Reasons for presentation to GP and psychiatrist

In relation to reasons for presentation to GPs and psychiatrists, rates varied substantially within onshore and offshore samples. For example, onshore and offshore between 0 and 62% of children presented with a general or unspecified complaint per quarter, while between 0 and 26% of children presented with a complaint about psychological issues per quarter (Table S4, Supporting Information). Our method suggested that complaints related to skin (*z* = −1.97; *P* = 0.05), respiratory issues (*z* = −1.96; *P* = 0.05) and urological issues (*z* = −2.21; *P* = 0.03) were significantly higher amongst children detained offshore (Table 2). Between 10 and 40% of children presented with some type of respiratory or skin complain per quarter offshore, with rates about two to three times higher on average offshore. Between 0 and 31% of children presented with urological complaints per quarter offshore, with rates for urological complaints about one and half times higher offshore per quarter (Table S4, Supporting Information). While no other significant differences were found, a similar pattern can be seen across other variables, with offshore rates generally higher. Median rates per quarter, *z* and *P* values, are summarised in Table 2.

**Table 2:** Reasons for presentation to general practitioner and psychiatrist: Median events, range, *z* and *P* scores

Reason for presentation	Onshore	Offshore	Median *z* and *P* scores
	Median events/quarter (range)	Median events/quarter (range)	
General unspecified†	25.91 (0–50)	33.48 (7–62)	*z* = 1.63, *P* = 0.08
Psychological	14.38 (0–17)	10.93 (5–26)	*z* = 0.16, *P* = 0.26
Digestive	9.05 (0–21)	17.08 (5–33)	*z* = 1.30, *P* = 0.22
Skin	**10.62 (0–32)**	**23.26 (10–29)**	***z* = 1.97, *P* = 0.05**
Musculoskeletal	2.58 (0–8)	7.11 (2–12)	*z* = 1.35, *P* = 0.18
Respiratory	**10.29 (0–30)**	**26.95 (11–44)**	***z* = 1.96, *P* = 0.05**
Endocrine	7.32 (0–36)	5.16 (0–19)	*z* = 0.47, *P* = 0.51
Cardiovascular	0.58 (0–3)	1.66 (0–4)	*z* = 0.46, *P* = 0.47
Eye	2.01 (0–5)	3.04 (0–8)	*z* = 0.66, *P* = 0.46
Social	11.12 (0–25)	16.01 (0–33)	*z* = 0.36, *P* = 0.47
Neurological	1.53 (0–5)	2.87 (0–6)	*z* = 73, *P* = 0.47
Blood	1.09 (0–8)	0.74 (0–6)	*z* = 0.02, *P* = 0.45
Ear	3.33 (0–9)	6.56 (0–13)	*z* = −0.96, *P* = 0.32
Urological	**4.76 (0–12)**	**11.02 (0–31)**	***z* = 2.21, *P* = 0.03**
Pregnancy	0.15 (0–2)	0.00 (0)	*z* = 0.71, *P* = 0.47
Genital	1.35 (0–6)	0.00 (0–5)	*z* = 0.78, *P* = 0.32
Injury	0.58 (0–7)	6.67 (2–10)	*z* = 1.07, *P* = 0.29

† General unspecified includes a wide range of non‐specific presentations such as those associated with viral infections.

The bold values indicate the significant results.

### Consultations by health professional

In relation to number of consultations by health professionals, our method suggested that children offshore had significantly higher rates of consultations with a mental health nurse (*z* = −1.96; *P* = 0.002), psychologist (*z* = −2.32; *P* = 0.01) and counsellor (*z* = −3.41; *P* < 0.001) (Table 3). Offshore between 40 and 70% of children saw a GP per quarter. Between 20 and 58% of children saw a mental health nurse per quarter offshore, while between 4 and 50% saw a psychologist, with rates of consultations for psychological support about 1.5 times higher offshore (Table S5, Supporting Information). Between 0 and 33% of children saw a counsellor per quarter offshore, with these rates up to 16 times higher on average than those onshore. Both GP and nurse appointments were also notably higher offshore, however, did not meet our threshold of significance (while median *P* values were <0.05, median *z* values were not ±1.96). These results are more difficult to interpret as access to GP and nurse appointments appeared to fluctuate substantially between onshore and offshore detention. Data suggest that between 25 and 100% of children saw a nurse both onshore and offshore per quarter, while 6–96% of children saw a GP onshore and offshore every quarter. Median rates per quarter, *z* and *P* values are summarised in Table 3.

**Table 3:** Consultations by health professional: Median events, range, *z* and *P* scores

	Onshore	Offshore	Median *z* and *P* scores
	Median events/quarter (range)	Median events/quarter (range)	
GP	49.28 (6–80)	64.65 (26–96)	*z* = −1.62, *P* = 0.05
RN	85.94 (43–97)	81.92 (27–137)	*z* = −1.35, *P* < 0.001
MHN	**40.03 (0–75)**	**52.73 (24–85)**	***z* = −1.96, *P* = 0.002**
Psychologist	**17.65 (0–46)**	**25.54 (4–97)**	***z* = −2.32, *P* = 0.01**
Counsellor	**2.81 (0–34)**	**32.22 (3–63)**	***z* = −3.41, *P* < 0.001**
Psychiatrist	8.42 (0–16)	8.53 (0–47)	*z* = −0.72, *P* = 0.19

GP, general practitioner; MHN, mental health nurse; RN, registered nurse.

The bold values indicate the significant results.

### Prescribed medications

For prescribed medication rates again generally appeared to be higher offshore, however, our method revealed this was only significant for 3 drug types, with penicillin (*z* = −3.10; *P* < 0.001), antihistamine (*z* = −3.66; *P* < 0.001) and expectorant (*z* = −3.56; *P* < 0.001) prescriptions significantly higher offshore (Table 4). Between 9 and 26% of children were prescribed penicillin offshore per quarter, which was on average about 3 times higher than rates onshore (Table S6, Supporting Information). Between 4 and 27% of children offshore were prescribed antihistamines per quarter, which was on average about 4 times the rate of children onshore. And between 2 and 28% of children were prescribed expectorants per quarter offshore, which was on average about 4 times higher than the rate per quarter onshore. Median rates per quarter, *z* and *P* values are summarised in Table 4.

**Table 4:** Prescribed medication: Median events, range, *z* and *P* scores

Prescription class	Onshore	Offshore	Median z and *P* scores
	Median events/quarter (range)	Median events/quarter (range)	
NSAIDS	7.69 (3–16)	14.50 (5–25)	*z* = −1.42, *P* = 0.15
Analgesics	24.84 (3–53)	29.98 (11–53)	*z* = −1.32, *P* = 0.15
Hyperacidity, reflux and ulcers	2.73 (1–5)	3.32 (2–9)	*z* = −0.73, *P* = 0.27
Antidepressants	3.72 (2–8)	2.22 (1–5)	*z* = 0.86, *P* = 0.37
Antipsychotics	1.24 (0–6)	2.13 (1–2)	*z* = −0.38, *P* = 0.39
Penicillin	**6.45 (5–17)**	**16.43 (9–26)**	***z* = −3.10, *P* < 0.001**
Antihistamines	**3.94 (2–8)**	**14.23 (4–27)**	***z* = −3.66, *P* < 0.001**
Topical antifungals	0.88 (1–8)	5.54 (3–11)	*z* = −1.83, *P* = 0.06
Topical corticosteroids	4.56 (1–15)	6.67 (3–14)	*z* = 1.14, *P* = 0.13
Expectorants	**1.37 (1–2)**	**6.99 (2–28)**	***z* = −3.56, *P* < 0.001**

NSAIDS: non steroidal anti inflammatory drugs.

The bold values indicate the significant results.

### Specialist referrals, chronic disease, disability and torture and trauma disclosures

The quarterly health reports also contain a number of other variables such as specialist referrals and psychiatric hospital admissions. Because these events were far less common, no comparison could be made; however, these numbers suggest that a small number of particularly vulnerable children were detained onshore and offshore between 2014 and 2017. Results are summarised in Table 5.

**Table 5:** Number of specialist referrals, chronic disease, disability and torture and trauma disclosures reported onshore and offshore for all quarters

	Specialist referrals	Chronic disease presentations	Disability presentations	Torture and trauma disclosures	Psychiatric hospital admissions
Onshore (Q3 2014–Q4 2018)	74	138	121	95	7
Offshore (Q3 2014–Q4 2017)	2	63	11	5	0

## Discussion

The above results suggest that children detained both onshore and offshore have substantial health needs and that a significant number both on and offshore are likely to have had health problems while detained. Children offshore presented more frequently with a range of complaints and accessed health‐care professionals at higher rates. Of additional concern, a small but substantial number of children were recorded with disabilities and chronic illness, a number required specialist intervention, some children disclosed torture and trauma and seven children were admitted to a psychiatric hospital in Australia. It is likely that specialist referrals, chronic disease, disability and hospital admissions were higher onshore because many unwell children have been evacuated from Nauru^11^ and furthermore, Nauru has few facilities for paediatric or psychiatric care. Torture and trauma disclosures are likely underreported here as majority of those seeking asylum often disclose shortly after arrival, and subsequent disclosures are not counted in the quarterly reports.

How do these results compare to past studies and children in the Australian community? While more direct comparisons are difficult to make, Australian community data sheds more light on the above results. In the Australian community, 60–70% of children see a GP at least once annually.^12^ In our sample, up to 80% of children onshore and 96% of children offshore saw a GP per quarter. In regards to mental health services, 17% of children in the Australian community access at least one mental health service annually; of those with a diagnosed mental health condition, 56% access at least one service annually, with 24% accessing a psychologist, 3% accessing a nurse, 7% accessing a psychiatrist and 21% accessing a counsellor.^13^ Rates were higher amongst our sample each quarter, up to 75% (onshore) to 100% (offshore) of children saw a mental health nurse, 46% (onshore) to 97% (offshore) of children saw a psychologist and 34% (onshore) to 63% (offshore) of children saw a counsellor (Table S5, Supporting Information). Finally, approximately 1.8% of Australians aged 0–17 years were prescribed antidepressants between 2017 and 2018^14^ compared with up to 8% of the population onshore and 5% of the population offshore being prescribed antidepressants. While we could not run direct comparisons here, it is clear that children detained in onshore and offshore immigration detention have higher physical and mental health‐care needs than children in the general population.

There are several limitations that should be noted. First, a degree of selection bias is acknowledged in the quarterly health reports; those who had been detained for protracted periods or those with grievances in relation to the health care provider or staff were less likely to engage with health services (this was specifically noted as an issue in a number of the quarterly health reports). This would have led to an underestimation reported above of the health care needs, particularly of children in offshore detention. Second, it is not known if the data was collected reliably and consistently. Third, while our data clearly demonstrate children in immigration detention and particularly offshore detention have very high health‐care needs, a cause and effect relationship in regards to the impact of detention on the health of children cannot be determined. Further caution is warranted in relation to the statistical analyses we applied, we were somewhat limited in that we only had access to aggregated data, where assumptions of independence were violated. Fourth, a further explanation as to why differences were or were not observed related to the availability of health care in offshore detention, and there may simply have been greater access to health care offshore versus onshore, although this is unlikely. As noted in the introduction, there is substantial evidence so suggest that Nauru (offshore) had limited facilities, particularly for paediatric care. A final limitation relates more to the quarterly health reports themselves; in many ways, these reports say very little. Better reporting of health information should be made a priority to enable a transparent understanding of the health and wellbeing of detained children.

## Conclusion

The results of this study suggest that children detained in Australian immigration detention centres have far greater health needs than those in the Australian community. Children in detention centres access health‐care services at high rates are prescribed medication at high rates and are experiencing social, emotional and behavioural problems as evidenced by their attendance for psychological support. Health problems appear to be greater in offshore detention, with health complaints more frequently reported, health‐care professionals accessed far more regularly and medications prescribed far more often. Furthermore, a small but particularly concerning group of children appear to have been detained, with hundreds detained between 2014 and 2018 reporting disabilities, chronic illness and past torture and trauma.

## Supporting information


**Appendix** **S1**. Detailed methods.
**Table S1**. Number of events per quarter: Reasons for presentation to GP/psychiatrist.
**Table S2**. Number of events per quarter: Appointments by health professional.
**Table S3**. Number of events per quarter: Number of children who were prescribed medication.
**Table S4**. Rate per 100 children per quarter: Reasons for presentation to GP/Psychiatrist.
**Table S5**. Rate per 100 children per quarter: Appointments by health professional.
**Table S6**. Rate per 100 children per quarter: Prescribed medication.
