# Supplementary material for: Health of children in Australian immigration detention centres: An analysis of the quarterly health reports from 2014 to 2017
Source: J Paediatr Child Health. 2022 Jan 18;58(6):985–90. doi: 10.1111/jpc.15880 (PMC9305240; doi:10.1111/jpc.15880)
Supplement: Supplementary file 1 — Appendix S1. Detailed methods. Table S1. Number of events per quarter: Reasons for presentation to GP/psychiatrist. Table S2. Number of events per quarter: Appointments by health professional. Table S3. Number of events per quarter: Number of children who were prescribed medication. Table S4. Rate per 100 children per quarter: Reasons for presentation to GP/Psychiatrist. Table S5. Rate per 100 children per quarter: Appointments by health professional. Table S6. Rate per 100 children per quarter: Prescribed medication. [file JPC-58-985-s001.docx]

Detailed Methods

Data Sources

In this study we utilised the Australian government’s Quarterly Immigration Detention Health Reports over a period of 3 years (from Quarter 3, 2014, to Quarter 2 2017) for onshore and offshore detention^^[[1]](#footnote-1)^^. These reports are produced by the immigration detention healthcare provider, International Health and Medical Services (IHMS) and provided to the Australian government quarterly. These reports contain data about the health and wellbeing of children, including complaints/presenting symptoms and number of appointments and hospitalisations, among other variables. These reports were either already publicly available^^[[2]](#footnote-2)^^ or obtained through Freedom of Information Requests sent to the Australian Department of Home Affairs.

Data entry, transformation and the detention population

Data were entered manually by two authors (RE and EK), screened and cleaned. A number of issues were noted where data were recorded inconsistently in the quarterly reports. Where possible, mistakes and omissions were recalculated by the authors and data were replaced. If this was not possible data were excluded from further analysis.

The detention population

To compare onshore and offshore datasets, the data were transformed. To do this, we first had to estimate the quarterly detention population of children. Childhood and adolescence was not defined in the reports but is assumed to include children under 18 years of age in this study. The detention population was informed by the monthly Australian immigration detention population statistics, (again publicly available and published by the Australian government)^^[[3]](#footnote-3)^^ and the above quarterly health reports. For those detained onshore, while the department of home affairs publishes monthly statistical reports on the immigration detention population, these data are cross sectional; it remains difficult to determine the number of new and released detainees on a quarter by quarter basis. Every month, statistics reveal that in onshore detention, hundreds of people are detained for less than 3 months, many also remain detained for multiple quarters. This means relying solely on monthly (cross-sectional) immigration detention reports would lead to significant overestimates in the number of appointments, diagnoses or events per person in onshore detention centres. A more accurate figure was calculated by using the quarterly health reports. For example, a number of tables in the quarterly health reports state the percentage reported of the population prescribed medication and the number of individuals (n)prescribed medication for the quarter. From this, we estimated the total number of children in onshore detention using the formulae (i.e. $\frac{n\times100}{\frac{0}{0}reported}$ ). To calculate the offshore population we utilised the cross-sectional population of children, this is because after mid 2014 the population was largely static, that is, few people would have moved in and out within the quarter. We did explore whether the above method (that was applied to onshore data) would make any significant difference to our results, and we found that cross sectional data were generally accurate with the calculated percentages reported from the offshore quarterly health reports to within 1-2 percent in most cases. The calculated total populations of children in onshore and offshore detention centres per quarter are reported below in tables 1-3 along with the number of health events that were contained in the quarterly reports.

Rates for onshore and offshore detention

After calculating the detention population for both onshore and offshore data, we estimated the rate of health events per quarter against the quarterly onshore or offshore detention population. Health related events were converted to rates per 100 child detainees per quarter (i.e. $p= \frac{e}{n}\times100$). These rates allowed us to make a more direct comparison between onshore and offshore health events. We have relied on data reporting ‘unique individuals’ as opposed to ‘unique appointments’ per quarter. That is, rates reported below reflect the number of children per quarter that (on average) accessed services or were prescribed medication for example. Rates of health events are reported in tables 4-6.

Comparing onshore and offshore events

Given the detention population, and because quarter by quarter many of the same children were detained, particularly for the offshore population, this data violated assumptions for independence of observations, limiting the significance tests that could be carried out. To overcome this, we opted to run a series of two-proportion z-tests for each matched quarter between onshore and offshore data (2014 Q3 to 2017 Q2). To do this we used the below formula where $n_{A}$ and $n_{B}$ = the detention population; $pA$ and $pB$ = the number of health events; and where $p$ and $q$ = the overall proportions (i.e. $p$ = ($pA+pB)/{(n}_{A}+n_{B})$ and $q=1- p$.

$$z=\frac{pA-pB}{\sqrt{pq/n_{A}+pq/}n_{B}}$$

After calculating a z and p value for each quarter, we calculated median z and p values for all quarters and utilised this as an indicator as to whether the observed differences between onshore and offshore events were statistically significant. We opted to use median scores because of the relatively small sample size (i.e. 12 quarters) and because of the skew of some of these variables. Furthermore, as we were using median scores and in taking a relatively conservative approach, we have only considered a result significant when both z = ± 1.96 and p < .05.

Ethics approval

Ethics approval for this study was granted by the University of Greenwich, Human Research Ethics Committee (UREC/20.1.5.6).

Variables

Reasons for presentation to GP and Psychiatrist

The reasons why children presented to GPs and Psychiatrists were recorded consistently in all reports. This data cannot be considered diagnoses, but more as presenting complaints or symptoms. These presentations also represent a wide range of symptoms. For example, cases captured under the “psychological” grouping range from recognised psychiatric diagnoses, to psychologically related consults such as smoking cessation activities. For all reports we used the total number of unique children who had attended for each issue. That is, if a child attended twice for the same issue, this would have only been recorded once.

Number of consultations by health profession

The number of children who presented to a range of healthcare professionals (GPs, Psychiatrists, Nurses, Psychologists and Counsellors) were also reported across a number of years. For all reports we used the number of unique children who had attended for each type of health professional. Again, if a child attended two or more times to the same professional, this would have only been recorded once, however the same child could present with the same condition again the next quarter or for a different condition in the same quarter. Overall rates for children were not available, so we could not gauge if children had attended two or more times for the same type of professional.

Prescribed medications

The twenty most commonly prescribed medications were reported in all reports. Data captured the total number of unique children who received prescription medication.

Specialist referrals, chronic disease, disability and torture and trauma disclosures

The quarterly health reports also include a range of other variables related to health and health outcomes. While these generally represent a small number of individuals, we have included them here to illustrate that hundreds of particularly vulnerable children were detained between 2014-2018. The variables reported include, the number of children who had presented each quarter with a chronic condition or a disability (not children who were diagnosed with a disability/chronic condition or the total number of children detained with a disability/chronic condition), number of specialist referrals, number of torture and trauma disclosures and psychiatric hospital admissions.

# Raw data: Detention population, events and rates

Supplementary table 1. Number of events per quarter: Reasons for presentation to GP/Psychiatrist

|  | Onshore population | General unspecified | Psychological | Digestive | Skin | Musculoskeletal | Respiratory | Endocrine | Cardiovascular | Eye | Social | Neurological | Blood | Ear | Urological | Pregnancy | Genital | Injury | Total |
| --- | --- | --- | --- | --- | --- | --- | --- | --- | --- | --- | --- | --- | --- | --- | --- | --- | --- | --- | --- |
| 2014 Q3 | 700 | 289 | 112 | 121 | 229 | 48 | 211 | 254 | 12 | 31 | 100 | 31 | 59 | 61 | 84 | 1 | 25 | 49 | 1717 |
| 2014 Q4 | 622 | 214 | 90 | 105 | 110 | 30 | 128 | 110 | 9 | 30 | 74 | 16 | 37 | 37 | 73 | 1 | 15 | 19 | 1098 |
| 2015 Q1 | 455 | 99 | 65 | 35 | 44 | 11 | 41 | 43 | 14 | 10 | 47 | 7 | 13 | 0 | 18 | 2 | 4 | 16 | 469 |
| 2015 Q2 | 173 | 52 | 28 | 18 | 20 | 10 | 20 | 12 | 2 | 4 | 24 | 4 | 6 | 7 | 4 | 1 | 5 | 2 | 219 |
| 2015 Q3 | 153 | 64 | 17 | 21 | 24 | 7 | 26 | 17 | 2 | 6 | 26 | 5 | 1 | 6 | 10 | 3 | 1 | 2 | 238 |
| 2015 Q4 | 132 | 66 | 20 | 28 | 21 | 1 | 24 | 9 | 2 | 4 | 30 | 2 | 2 | 6 | 13 | 2 | 3 | 0 | 233 |
| 2016 Q1 | 110 | 47 | 17 | 23 | 14 | 3 | 20 | 9 | 0 | 2 | 28 | 5 | 2 | 3 | 13 | 2 | 2 | 0 | 190 |
| 2016 Q2 | 32 | 2 | 1 | 0 | 0 | 0 | 2 | 0 | 0 | 0 | 0 | 0 | 0 | 0 | 0 | 0 | 0 | 0 | 5 |
| 2016 Q3 | 13 | 0 | 0 | 0 | 0 | 0 | 0 | 0 | 0 | 0 | 0 | 0 | 0 | 0 | 0 | 0 | 0 | 0 | 0 |
| 2016 Q4 | 13 | 0 | 1 | 0 | 0 | 1 | 0 | 1 | 0 | 0 | 0 | 0 | 0 | 0 | 0 | 0 | 0 | 0 | 3 |
| 2017 Q1 | 33 | 0 | 1 | 0 | 0 | 0 | 0 | 1 | 0 | 0 | 0 | 0 | 0 | 0 | 0 | 0 | 0 | 0 | 2 |
| 2017 Q2 | 18 | 0 | 3 | 0 | 0 | 0 | 1 | 0 | 0 | 0 | 0 | 0 | 0 | 1 | 1 | 0 | 1 | 1 | 8 |
| Total | 2454 | 833 | 355 | 351 | 462 | 111 | 473 | 456 | 41 | 87 | 329 | 70 | 120 | 121 | 216 | 12 | 56 | 89 | 4182 |
|  | Offshore population |  |  |  |  |  |  |  |  |  |  |  |  |  |  |  |  |  |  |
| 2014 Q3 | 186 | 60 | 28 | 47 | 48 | 22 | 54 | 18 | 4 | 11 | 17 | 8 | 4 | 12 | 36 | 0 | 3 | 17 | 389 |
| 2014 Q4 | 135 | 84 | 32 | 44 | 37 | 9 | 59 | 25 | 5 | 5 | 28 | 5 | 8 | 18 | 32 | 0 | 4 | 12 | 407 |
| 2015 Q1 | 103 | 43 | 27 | 18 | 16 | 7 | 36 | 8 | 2 | 5 | 34 | 6 | 3 | 11 | 23 | 0 | 5 | 7 | 251 |
| 2015 Q2 | 88 | 28 | 15 | 9 | 9 | 3 | 17 | 3 | 0 | 1 | 12 | 1 | 0 | 7 | 6 | 0 | 2 | 2 | 115 |
| 2015 Q3 | 92 | 49 | 6 | 10 | 25 | 8 | 23 | 6 | 2 | 2 | 17 | 5 | 2 | 5 | 14 | 0 | 0 | 2 | 176 |
| 2015 Q4 | 68 | 30 | 4 | 13 | 20 | 7 | 22 | 4 | 1 | 3 | 17 | 1 | 1 | 4 | 20 | 0 | 0 | 6 | 153 |
| 2016 Q1 | 54 | 31 | 7 | 9 | 13 | 4 | 18 | 9 | 1 | 1 | 11 | 2 | 0 | 7 | 17 | 0 | 0 | 1 | 131 |
| 2016 Q2 | 49 | 17 | 8 | 10 | 11 | 4 | 11 | 0 | 1 | 4 | 9 | 1 | 0 | 3 | 1 | 0 | 0 | 2 | 82 |
| 2016 Q3 | 45 | 5 | 3 | 8 | 12 | 1 | 13 | 1 | 0 | 2 | 1 | 0 | 0 | 3 | 0 | 0 | 1 | 3 | 53 |
| 2016 Q4 | 45 | 5 | 4 | 3 | 6 | 4 | 10 | 2 | 0 | 0 | 0 | 0 | 1 | 3 | 1 | 0 | 0 | 3 | 42 |
| 2017 Q1 | 45 | 3 | 4 | 4 | 10 | 3 | 5 | 0 | 0 | 1 | 0 | 2 | 0 | 0 | 2 | 0 | 0 | 3 | 37 |
| 2017 Q2 | 42 | 7 | 2 | 2 | 5 | 2 | 6 | 1 | 0 | 1 | 0 | 0 | 0 | 1 | 0 | 0 | 0 | 4 | 31 |
| Total | 952 | 362 | 140 | 177 | 212 | 74 | 274 | 77 | 16 | 36 | 146 | 31 | 19 | 74 | 152 | 0 | 15 | 62 | 1867 |

Supplementary table 2. Number of events per quarter: Appointments by health professional

|  | Onshore Population | GP | RN | MHN | Psychologist | Counsellor | Psychiatrist | Total |
| --- | --- | --- | --- | --- | --- | --- | --- | --- |
| 2014 Q3 | 700 | 561 | 680 | 528 | 325 | 235 | 110 | 2535 |
| 2014 Q4 | 622 | 399 | 573 | 362 | 126 | 126 | 90 | 1716 |
| 2015 Q1 | 455 | 183 | 356 | 154 | 82 | 46 | 59 | 880 |
| 2015 Q2 | 173 | 84 | 148 | 83 | 38 | 5 | 21 | 379 |
| 2015 Q3 | 153 | 94 | 132 | 74 | 32 | 1 | 14 | 347 |
| 2015 Q4 | 132 | 98 | 113 | 61 | 34 | 5 | 10 | 321 |
| 2016 Q1 | 110 | 66 | 98 | 55 | 19 | 3 | 15 | 256 |
| 2016 Q2 | 32 | 2 | 14 | 2 | 2 | 0 | 1 | 21 |
| 2016 Q3 | 13 | 3 | 13 | 0 | 0 | 0 | 0 | 16 |
| 2016 Q4 | 13 | 2 | 10 | 1 | 0 | 0 | 1 | 14 |
| 2017 Q1 | 33 | 7 | 22 | 1 | 0 | 0 | 1 | 31 |
| 2017 Q2 | 18 | 9 | 18 | 2 | 0 | 1 | 1 | 31 |
| Total | 2454 | 1508 | 2177 | 1323 | 658 | 422 | 323 | 6547 |
|  | Offshore Population | GP | RN | MHN | Psychologist | Counsellor | Psychiatrist | Total |
| 2014 Q3 | 186 | 132 | 193 | 111 | 180 | 36 | 42 | 726 |
| 2014 Q4 | 135 | 130 | 162 | 82 | 51 | 39 | 21 | 532 |
| 2015 Q1 | 103 | 68 | 141 | 74 | 51 | 44 | 31 | 468 |
| 2015 Q2 | 88 | 39 | 79 | 26 | 24 | 29 | 16 | 213 |
| 2015 Q3 | 92 | 62 | 86 | 50 | 4 | 36 | 43 | 281 |
| 2015 Q4 | 68 | 47 | 73 | 23 | 11 | 2 | 0 | 156 |
| 2016 Q1 | 54 | 38 | 40 | 46 | 25 | 17 | 2 | 168 |
| 2016 Q2 | 49 | 31 | 36 | 33 | 16 | 31 | 4 | 151 |
| 2016 Q3 | 45 | 25 | 23 | 23 | 8 | 24 | 0 | 103 |
| 2016 Q4 | 45 | 21 | 15 | 23 | 4 | 16 | 4 | 83 |
| 2017 Q1 | 45 | 19 | 12 | 15 | 7 | 13 | 3 | 69 |
| 2017 Q2 | 42 | 11 | 12 | 10 | 10 | 7 | 1 | 51 |
| Total | 952 | 623 | 872 | 516 | 391 | 294 | 167 | 3001 |

Note. Total does not reflect the numbers presented in this table as this table only presents the most common appointments. Total numbers also include physiotherapy and paramedic appointments from Q3 2014-Q1 2015 – these were not included in the above analysis as this was only reported over three quarters.

Supplementary table 3. Number of events per quarter: Number of children who were prescribed medication

|  | Onshore Population | NSAIDS | Analgesics | Hyperacidity, reflux and ulcers | Antidepressants | Antipsychotics | Penicillin | Antihistamines | Topical antifungals | Topical corticosteroids | Expectorants | Total |
| --- | --- | --- | --- | --- | --- | --- | --- | --- | --- | --- | --- | --- |
| 2014 Q3 | 700 | 103 | 371 | 18 | 18 | 6 | 116 | 42 |  | 32 | 11 | 1164 |
| 2014 Q4 | 622 | 101 | 262 | 16 | 14 | 3 | 67 | 31 |  | 30 | 6 | 723 |
| 2015 Q1 | 455 | 25 | 113 | 4 | 11 | 6 | 22 | 8 | 4 | 11 |  | 235 |
| 2015 Q2 | 173 | 12 | 46 | 5 | 5 | 2 | 11 | 5 | 1 | 4 | 2 | 110 |
| 2015 Q3 | 153 | 15 | 36 | 8 | 7 | 2 | 10 | 4 | 4 | 7 |  | 114 |
| 2015 Q4 | 132 | 13 | 44 | 6 | 10 | 4 | 7 | 7 | 1 | 6 | 3 | 122 |
| 2016 Q1 | 110 | 13 | 40 | 3 | 5 | 1 | 6 | 2 | 3 | 1 |  | 87 |
| 2016 Q2 | 32 | 1 | 2 |  |  |  |  |  |  |  |  | 3 |
| 2016 Q3 | 13 |  |  |  |  |  |  |  |  | 2 |  | 2 |
| 2016 Q4 | 13 | 1 | 1 |  |  |  |  | 1 |  |  |  | 3 |
| 2017 Q1 | 33 | 1 | 1 |  |  |  |  |  |  |  |  | 2 |
| 2017 Q2 | 18 | 1 | 3 |  | 1 | 1 | 2 |  |  |  |  | 11 |
| Total | 2454 | 286 | 919 | 60 | 71 | 25 | 241 | 100 | 13 | 93 | 22 | 2576 |
|  | Offshore population | NSAIDS | Analgesics | Hyperacidity, reflux and ulcers | Antidepressants | Antipsychotics | Penicillin | Antihistamines | Topical antifungals | Topical corticosteroids | Expectorants | Total |
| 2014 Q3 | 186 | 34 | 79 | 17 | 9 |  | 44 | 41 | 12 | 5 | 13 | 301 |
| 2014 Q4 | 135 | 34 | 66 | 5 | 1 | 1 | 35 | 37 | 7 | 5 | 12 | 228 |
| 2015 Q1 | 103 | 24 | 55 | 3 | 3 | 2 | 21 | 22 | 3 |  | 17 | 173 |
| 2015 Q2 | 88 | 13 | 23 | 5 | 1 |  | 19 | 12 | 3 |  | 5 | 110 |
| 2015 Q3 | 92 | 12 | 27 | 2 | 0 | 0 | 18 | 14 | 7 |  | 8 | 110 |
| 2015 Q4 | 68 | 10 | 34 | 2 |  |  | 11 | 11 | 4 |  | 19 | 126 |
| 2016 Q1 | 54 | 3 | 20 | 1 | 1 | 1 | 9 | 8 |  | 5 | 6 | 77 |
| 2016 Q2 | 49 | 7 | 15 | 3 | 1 | 1 | 7 | 5 | 2 | 7 | 2 | 72 |
| 2016 Q3 | 45 | 6 | 7 | 2 | 2 | 1 | 7 | 6 | 5 | 2 | 2 | 60 |
| 2016 Q4 | 45 | 9 | 13 |  | 1 | 1 | 4 | 5 | 2 | 3 | 1 | 47 |
| 2017 Q1 | 45 | 4 | 5 | 0 | 1 | 1 | 4 | 2 | 4 | 3 |  | 30 |
| 2017 Q2 | 42 | 2 | 9 | 1 | 1 | 1 | 5 | 3 |  |  | 1 | 28 |
| Total | 952 | 158 | 353 | 41 | 21 | 9 | 184 | 166 | 49 | 30 | 86 | 1362 |

Note. Total does not reflect the numbers presented in this table as this table only presents the most commonly prescribed medication. In reporting the number of prescriptions, quarterly reports only contained the ‘top 20’ prescribed medications per quarter. Thus, where medication was not reported therefore did not mean it was not prescribed that quarter. We opted to record this as missing as opposed to nil, which may have resulted in slight over-reporting of the mean and median rates of prescriptions.

Supplementary table 4. Rate per 100 children per quarter: Reasons for presentation to GP/Psychiatrist

| **Onshore** | **General unspecified** | **Psychological** | **Digestive** | **Skin** | **Musculo-skeletal** | **Respiratory** | **Endocrine** | **Cardiovascular** | **Eye** | **Social** | **Neurological** | **Blood** | **Ear** | **Urological** | **Pregnancy** |  | **Genital** | **Injury** |
| --- | --- | --- | --- | --- | --- | --- | --- | --- | --- | --- | --- | --- | --- | --- | --- | --- | --- | --- |
| 2014 Q3 | 41.29 | 16.00 | 17.29 | 32.71 | 6.86 | 30.14 | 36.29 | 1.71 | 4.43 | 14.29 | 4.43 | 8.43 | 8.71 | 12.00 | 0.14 |  | 3.57 | 7.00 |
| 2014 Q4 | 34.41 | 14.47 | 16.88 | 17.68 | 4.82 | 20.58 | 17.68 | 1.45 | 4.82 | 11.90 | 2.57 | 5.95 | 5.95 | 11.74 | 0.16 |  | 2.41 | 3.05 |
| 2015 Q1 | 21.76 | 14.29 | 7.69 | 9.67 | 2.42 | 9.01 | 9.45 | 3.08 | 2.20 | 10.33 | 1.54 | 2.86 | 0.00 | 3.96 | 0.44 |  | 0.88 | 3.52 |
| 2015 Q2 | 30.06 | 16.18 | 10.40 | 11.56 | 5.78 | 11.56 | 6.94 | 1.16 | 2.31 | 13.87 | 2.31 | 3.47 | 4.05 | 2.31 | 0.58 |  | 2.89 | 1.16 |
| 2015 Q3 | 41.83 | 11.11 | 13.73 | 15.69 | 4.58 | 16.99 | 11.11 | 1.31 | 3.92 | 16.99 | 3.27 | 0.65 | 3.92 | 6.54 | 1.96 |  | 0.65 | 1.31 |
| 2015 Q4 | 50.00 | 15.15 | 21.21 | 15.91 | 0.76 | 18.18 | 6.82 | 1.52 | 3.03 | 22.73 | 1.52 | 1.52 | 4.55 | 9.85 | 1.52 |  | 2.27 | 0.00 |
| 2016 Q1 | 42.73 | 15.45 | 20.91 | 12.73 | 2.73 | 18.18 | 8.18 | 0.00 | 1.82 | 25.45 | 4.55 | 1.82 | 2.73 | 11.82 | 1.82 |  | 1.82 | 0.00 |
| 2016 Q2 | 6.25 | 3.13 | 0.00 | 0.00 | 0.00 | 6.25 | 0.00 | 0.00 | 0.00 | 0.00 | 0.00 | 0.00 | 0.00 | 0.00 | 0.00 |  | 0.00 | 0.00 |
| 2016 Q3 | 0.00 | 0.00 | 0.00 | 0.00 | 0.00 | 0.00 | 0.00 | 0.00 | 0.00 | 0.00 | 0.00 | 0.00 | 0.00 | 0.00 | 0.00 |  | 0.00 | 0.00 |
| 2016 Q4 | 0.00 | 7.69 | 0.00 | 0.00 | 7.69 | 0.00 | 7.69 | 0.00 | 0.00 | 0.00 | 0.00 | 0.00 | 0.00 | 0.00 | 0.00 |  | 0.00 | 0.00 |
| 2017 Q1 | 0.00 | 3.03 | 0.00 | 0.00 | 0.00 | 0.00 | 3.03 | 0.00 | 0.00 | 0.00 | 0.00 | 0.00 | 0.00 | 0.00 | 0.00 |  | 0.00 | 0.00 |
| 2017 Q2 | 0.00 | 16.67 | 0.00 | 0.00 | 0.00 | 5.56 | 0.00 | 0.00 | 0.00 | 0.00 | 0.00 | 0.00 | 5.56 | 5.56 | 0.00 |  | 5.56 | 5.56 |
| **Offshore** | **General unspecified** | **Psychological** | **Digestive** | **Skin** | **Musculo-skeletal** | **Respiratory** | **Endocrine** | **Cardiovascular** | **Eye** | **Social** | **Neurological** | **Blood** | **Ear** | **Urological** | **Pregnancy** |  | **Genital** | **Injury** |
| 2014 Q3 | 32.26 | 15.05 | 25.27 | 25.81 | 11.83 | 29.03 | 9.68 | 2.15 | 5.91 | 9.14 | 4.30 | 2.15 | 6.45 | 19.35 | 0.00 |  | 1.61 | 9.14 |
| 2014 Q4 | 62.22 | 23.70 | 32.59 | 27.41 | 6.67 | 43.70 | 18.52 | 3.70 | 3.70 | 20.74 | 3.70 | 5.93 | 13.33 | 23.70 | 0.00 |  | 2.96 | 8.89 |
| 2015 Q1 | 41.75 | 26.21 | 17.48 | 15.53 | 6.80 | 34.95 | 7.77 | 1.94 | 4.85 | 33.01 | 5.83 | 2.91 | 10.68 | 22.33 | 0.00 |  | 4.85 | 6.80 |
| 2015 Q2 | 31.82 | 17.05 | 10.23 | 10.23 | 3.41 | 19.32 | 3.41 | 0.00 | 1.14 | 13.64 | 1.14 | 0.00 | 7.95 | 6.82 | 0.00 |  | 2.27 | 2.27 |
| 2015 Q3 | 53.26 | 6.52 | 10.87 | 27.17 | 8.70 | 25.00 | 6.52 | 2.17 | 2.17 | 18.48 | 5.43 | 2.17 | 5.43 | 15.22 | 0.00 |  | 0.00 | 2.17 |
| 2015 Q4 | 44.12 | 5.88 | 19.12 | 29.41 | 10.29 | 32.35 | 5.88 | 1.47 | 4.41 | 25.00 | 1.47 | 1.47 | 5.88 | 29.41 | 0.00 |  | 0.00 | 8.82 |
| 2016 Q1 | 57.41 | 12.96 | 16.67 | 24.07 | 7.41 | 33.33 | 16.67 | 1.85 | 1.85 | 20.37 | 3.70 | 0.00 | 12.96 | 31.48 | 0.00 |  | 0.00 | 1.85 |
| 2016 Q2 | 34.69 | 16.33 | 20.41 | 22.45 | 8.16 | 22.45 | 0.00 | 2.04 | 8.16 | 18.37 | 2.04 | 0.00 | 6.12 | 2.04 | 0.00 |  | 0.00 | 4.08 |
| 2016 Q3 | 11.11 | 6.67 | 17.78 | 26.67 | 2.22 | 28.89 | 2.22 | 0.00 | 4.44 | 2.22 | 0.00 | 0.00 | 6.67 | 0.00 | 0.00 |  | 2.22 | 6.67 |
| 2016 Q4 | 11.11 | 8.89 | 6.67 | 13.33 | 8.89 | 22.22 | 4.44 | 0.00 | 0.00 | 0.00 | 0.00 | 2.22 | 6.67 | 2.22 | 0.00 |  | 0.00 | 6.67 |
| 2017 Q1 | 6.67 | 8.89 | 8.89 | 22.22 | 6.67 | 11.11 | 0.00 | 0.00 | 2.22 | 0.00 | 4.44 | 0.00 | 0.00 | 4.44 | 0.00 |  | 0.00 | 6.67 |
| 2017 Q2 | 16.67 | 4.76 | 4.76 | 11.90 | 4.76 | 14.29 | 2.38 | 0.00 | 2.38 | 0.00 | 0.00 | 0.00 | 2.38 | 0.00 | 0.00 |  | 0.00 | 9.52 |

Note: z and p values were unable to be calculated for total presentations as in many quarters the total number of presentations exceeded the number of children detained.

Supplementary table 5. Rate per 100 children per quarter: Appointments by health professional

| **Onshore** | **GP** | **RN** | **MHN** | **Psychologist** | **Counsellor** | **Psychiatrist** |
| --- | --- | --- | --- | --- | --- | --- |
| 2014 Q3 | 80.14 | 97.14 | 75.43 | 46.43 | 33.57 | 15.71 |
| 2014 Q4 | 64.15 | 92.12 | 58.20 | 20.26 | 20.26 | 14.47 |
| 2015 Q1 | 40.22 | 78.24 | 33.85 | 18.02 | 10.11 | 12.97 |
| 2015 Q2 | 48.55 | 85.55 | 47.98 | 21.97 | 2.89 | 12.14 |
| 2015 Q3 | 61.44 | 86.27 | 48.37 | 20.92 | 0.65 | 9.15 |
| 2015 Q4 | 74.24 | 85.61 | 46.21 | 25.76 | 3.79 | 7.58 |
| 2016 Q1 | 60.00 | 89.09 | 50.00 | 17.27 | 2.73 | 13.64 |
| 2016 Q2 | 6.25 | 43.75 | 6.25 | 6.25 | 0.00 | 3.13 |
| 2016 Q3 | 23.08 | 100.00 | 0.00 | 0.00 | 0.00 | 0.00 |
| 2016 Q4 | 15.38 | 76.92 | 7.69 | 0.00 | 0.00 | 7.69 |
| 2017 Q1 | 21.21 | 66.67 | 3.03 | 0.00 | 0.00 | 3.03 |
| 2017 Q2 | 50.00 | 100.00 | 11.11 | 0.00 | 5.56 | 5.56 |
| **Offshore** | **GP** | **RN** | **MHN** | **Psychologist** | **Counsellor** | **Psychiatrist** |
| 2014 Q3 | 70.97 | 103.76 | 59.68 | 96.77 | 19.35 | 22.58 |
| 2014 Q4 | 96.30 | 120.00 | 60.74 | 37.78 | 28.89 | 15.56 |
| 2015 Q1 | 66.02 | 136.89 | 71.84 | 49.51 | 42.72 | 30.10 |
| 2015 Q2 | 44.32 | 89.77 | 29.55 | 27.27 | 32.95 | 18.18 |
| 2015 Q3 | 67.39 | 93.48 | 54.35 | 4.35 | 39.13 | 46.74 |
| 2015 Q4 | 69.12 | 107.35 | 33.82 | 16.18 | 2.94 | 0.00 |
| 2016 Q1 | 70.37 | 74.07 | 85.19 | 46.30 | 31.48 | 3.70 |
| 2016 Q2 | 63.27 | 73.47 | 67.35 | 32.65 | 63.27 | 8.16 |
| 2016 Q3 | 55.56 | 51.11 | 51.11 | 17.78 | 53.33 | 0.00 |
| 2016 Q4 | 46.67 | 33.33 | 51.11 | 8.89 | 35.56 | 8.89 |
| 2017 Q1 | 42.22 | 26.67 | 33.33 | 15.56 | 28.89 | 6.67 |
| 2017 Q2 | 26.19 | 28.57 | 23.81 | 23.81 | 16.67 | 2.38 |

Note: z and p values were unable to be calculated for total appointments as in many quarters the total number of appointments exceeded the number of children detained.

Supplementary table 6. Rate per 100 children per quarter: Prescribed medication

| **Onshore** | **NSAIDS** | **Analgesics** | **Hyperacidity, reflux** | **Antidepressants** | **Antipsychotics** | **Penicillin** | **Antihistamines** | **Topical antifungals** | **Topical corticosteroids** | **Expectorants** |
| --- | --- | --- | --- | --- | --- | --- | --- | --- | --- | --- |
| 2014 Q3 | 14.71 | 53.00 | 2.57 | 2.57 | 0.86 | 16.57 | 6.00 |  | 4.57 | 1.57 |
| 2014 Q4 | 16.24 | 42.12 | 2.57 | 2.25 | 0.48 | 10.77 | 4.98 |  | 4.82 | 0.96 |
| 2015 Q1 | 5.49 | 24.84 | 0.88 | 2.42 | 1.32 | 4.84 | 1.76 | 0.88 | 2.42 |  |
| 2015 Q2 | 6.94 | 26.59 | 2.89 | 2.89 | 1.16 | 6.36 | 2.89 | 0.58 | 2.31 | 1.16 |
| 2015 Q3 | 9.80 | 23.53 | 5.23 | 4.58 | 1.31 | 6.54 | 2.61 | 2.61 | 4.58 |  |
| 2015 Q4 | 9.85 | 33.33 | 4.55 | 7.58 | 3.03 | 5.30 | 5.30 | 0.76 | 4.55 | 2.27 |
| 2016 Q1 | 11.82 | 36.36 | 2.73 | 4.55 | 0.91 | 5.45 | 1.82 | 2.73 | 0.91 |  |
| 2016 Q2 | 3.13 | 6.25 |  |  |  |  |  |  |  |  |
| 2016 Q3 |  |  |  |  |  |  |  |  | 15.38 |  |
| 2016 Q4 | 7.69 | 7.69 |  |  |  |  | 7.69 |  |  |  |
| 2017 Q1 | 3.03 | 3.03 |  |  |  |  |  |  |  |  |
| 2017 Q2 | 5.56 | 16.67 |  | 5.56 | 5.56 | 11.11 |  |  |  |  |
| **Offshore** | **NSAIDS** | **Analgesics** | **Hyperacidity, reflux** | **Antidepressants** | **Antipsychotics** | **Penicillin** | **Antihistamines** | **Topical antifungals** | **Topical corticosteroids** | **Expectorants** |
| 2014 Q3 | 18.28 | 42.47 | 9.14 | 4.84 |  | 23.66 | 22.04 | 6.45 | 2.69 | 6.99 |
| 2014 Q4 | 25.19 | 48.89 | 3.70 | 0.74 | 0.74 | 25.93 | 27.41 | 5.19 | 3.70 | 8.89 |
| 2015 Q1 | 23.30 | 53.40 | 2.91 | 2.91 | 1.94 | 20.39 | 21.36 | 2.91 |  | 16.50 |
| 2015 Q2 | 14.77 | 26.14 | 5.68 | 1.14 |  | 21.59 | 13.64 | 3.41 |  | 5.68 |
| 2015 Q3 | 13.04 | 29.35 | 2.17 |  |  | 19.57 | 15.22 | 7.61 |  | 8.70 |
| 2015 Q4 | 14.71 | 50.00 | 2.94 |  |  | 16.18 | 16.18 | 5.88 |  | 27.94 |
| 2016 Q1 | 5.56 | 37.04 | 1.85 | 1.85 | 1.85 | 16.67 | 14.81 |  | 9.26 | 11.11 |
| 2016 Q2 | 14.29 | 30.61 | 6.12 | 2.04 | 2.04 | 14.29 | 10.20 | 4.08 | 14.29 | 4.08 |
| 2016 Q3 | 13.33 | 15.56 | 4.44 | 4.44 | 2.22 | 15.56 | 13.33 | 11.11 | 4.44 | 4.44 |
| 2016 Q4 | 20.00 | 28.89 |  | 2.22 | 2.22 | 8.89 | 11.11 | 4.44 | 6.67 | 2.22 |
| 2017 Q1 | 8.89 | 11.11 |  | 2.22 | 2.22 | 8.89 | 4.44 | 8.89 | 6.67 |  |
| 2017 Q2 | 4.76 | 21.43 | 2.38 | 2.38 | 2.38 | 11.90 | 7.14 |  |  | 2.38 |

Note. Total does not reflect the rates presented in this table as this table only presents the most commonly prescribed medication; z and p values were unable to be calculated for total prescriptions as in many quarters the total number of prescriptions exceeded the number of children detained. In reporting the number of prescriptions, quarterly reports only contained the ‘top 20’ prescribed medications per quarter. Thus, where medication was not reported therefore did not mean it was not prescribed that quarter. We opted to record this as missing as opposed to nil, which may have resulted in slight over-reporting of the mean and median rates of prescriptions.

1. These reports are not available any earlier than these dates and the Australian government has not yet released reports beyond Q4 2017 offshore and Q4 2018 onshore. [↑](#footnote-ref-1)
2. https://www.homeaffairs.gov.au/access-and-accountability/freedom-of-information/disclosure-logs [↑](#footnote-ref-2)
3. https://www.homeaffairs.gov.au/research-and-statistics/statistics/visa-statistics/live/immigration-detention [↑](#footnote-ref-3)
